# The Foreign Journals: Midwifery

**Published:** 1839-01

**Authors:** 


### MIDWIFERY.

*Memoir on Absorption of the Placenta.* By Dr. Villeneuve, Chief Surgeon of the Hospice de la Maternité at Marseilles.

In the following memoir, Dr. Villeneuve brings forward several cases to prove that in certain instances of morbid adhesion of the placenta, the whole or part of that organ may be retained in the uterus, and be entirely absorbed into the system, without producing any serious effects on the constitution. In addition to two cases which he has himself met with, he notices six others which have been observed by different medical men, and which he relates at considerable length in this memoir. We will endeavour to extract, as fully as our limits will permit us, the principal facts in proof of this interesting occurrence.

Professor Naegele, of Heidelberg, appears to have been the first who called the attention of practitioners to the entire absorption of the placenta, without any dangerous results to the mother. He first observed the occurrence in 1803. Three cases collected by him are related in the present memoir, together with three others observed by Dr. Saloman, M. Paul Dubois, and M. Gabillot, which all nearly agree in the following particulars. In four out of six of them the labour was premature; in all of them there was unnatural adhesion of the placenta, either partial or total. The cases of partial adhesion, four in number, were attended with considerable hemorrhage, which, in two instances, was accompanied with irregular contraction of the uterus, impeding the introduction of the hand. In the other two cases the adhesion was so firm, that in one the cord broke off, and in the other it was found impossible to remove the whole of the placenta with the hand. In the cases of partial adhesion, also, considerable constitutional disturbance and uterine inflammation followed; though all the patients recovered: in none of the cases were any portions of the placenta expelled with the lochia. In the two instances where the adhesion was general, and the placenta not separated at all from the uterus, there was either a very trifling hemorrhage or none, and the placenta was totally absorbed without giving rise to any constitutional disturbance, and was accompanied with but very little lochial discharge.

In the first case observed by M. Villeneuve himself, the placenta was found morbidly adherent after a tedious labour, and was carefully detached, with the exception of a few fragments which could not be removed. The lochia were at first abundant and became very fœtid, but after a short time they were suppressed, and the patient sunk under typhus fever.

The author has reported his second case at considerable length. The patient, aged twenty-five, was confined at the end of the sixth month of her second pregnancy with three children. The placenta belonging to the second and third fœtuses were expelled directly after the birth of each child; but the afterbirth of the first infant the midwife could not succeed in extracting, and broke the cord. M. V. was called in; and on introducing his hand into the uterus, found the placenta firmly adherent by its whole circumference. The examination produced so much pain and irritation, that it was thought better not to attempt its removal, and the patient was taken to the Hospice de la Maternité, where she went on very favorably; the lochial discharge was very scanty. On examining the uterus externally on the eighth day, it was found reaching midway between the umbilicus and the pubes, and as large as in the fourth month of pregnancy. On the eleventh day, on examination by the vagina, the lower part of the uterus appeared occupied by some substance. The patient at this time returned home, and on the twentieth day from her confinement, subsequently to sexual intercourse, a considerable discharge took place resembling that of ordinary menstruation, only lasting for rather a longer time. No clot nor any solid substance was discharged. The patient was again examined on the thirty-first day from her confinement, when the uterus had returned to its natural size.

This case is remarkable in several respects, 1st, from being a triple birth; 2d, from the three placentæ being distinct, and one of them coming away between the birth of the second and third children; and, lastly, for the retention and absorption of the placenta, of which no portion was discharged or passed with the lochia, which were, indeed, less abundant than usual and not at all fœtid. What became of the first placenta is the most important question in this case; how can we account for its disappearance otherwise than by its absorption? Can we admit, with Mme. Boivin, the peculiar malformation of this organ, in which it is imperfectly developed and almost wanting? That idea cannot be adopted in the present instance, for the child belonging to the retained placenta lived longer than either of the others, and the umbilical cord was the largest of the three; so, judging from analogy, the retained placenta ought to have been as large if not superior in size to either of those extracted.

From the foregoing observations M. Villeneuve draws the following conclusions: 1. Absorption of the placenta, though an extraordinary fact, is nevertheless one quite incontestible. 2. This absorption can only take place, without producing lochial discharge or hemorrhage, where the adhesion is complete; and is accompanied with flooding when partial. 3. Total adhesion is perhaps never fatal: the cases of death only belong to those of partial adhesion, and arise either from hemorrhage, or by the absorption of putrid matter produced from the introduction of air, and the irritation of the uterus in the attempts to remove portions of the placenta, or, lastly, from the manner of interference producing metro-peritonitis. 4. A placenta which is not fixed to the uterus by organic and intimate adhesions cannot be absorbed, though it may perhaps be retained for several days without danger, if there is contraction of the uterus. 5. To stop the flooding, it is necessary to remove with the greatest care all the detached fragments in a case of partially adherent placenta. 6. Forcible separation of the placenta is both dangerous and useless; dangerous because the uterus may be injured, and useless because the adherent portions will be absorbed without increasing the danger of the case.

*Gazette Médicale de Paris. July* 8, 1837.

*Extra-Uterine Pregnancy occurring twice in succession in the same patient, at an interval of some years, and terminating favorably in both instances, and in the same manner.* By Dr. GALIAY.

A YOUNG woman shortly after being married was knocked down and ill treated in a quarrel. She was laid up for several days in consequence of the injuries which she received, but recovered without experiencing any further inconvenience at the time. Shortly after she found that she was pregnant, and her pregnancy seemed to follow the natural course. At the end of the time which she had calculated upon the usual symptoms of labour came on, but the pains went off without being followed by any result. Months passed, and she felt no other inconvenience, except her size remaining the same, and both her neighbours and herself began to doubt her having been pregnant at all. After a long interval, the exact duration of which Dr. Galiay was not acquainted with, she was seized with acute but intermitting pains in the abdomen, which were felt most severely in the neighbourhood of the groins and anus, accompanied with abdominal tenderness and febrile symptoms. The organs of generation were found on examination to be in their natural state. After a considerable period, a violent paroxysm of pain came on, accompanied with an immediate desire to evacuate the bowels, but her efforts to do so were fruitless. On examination, there was found the bone of a fœtus firmly impacted within the sphincter ani. After this was removed, she passed a number of others, which afforded her great relief, and she rapidly recovered her natural size and health. This happened in 1829. Subsequently to this she remained quite well till the year 1834, when she again became pregnant; and after some interval, the fragments of another fœtus were expelled per anum as at the former time, the only remarkable difference being that in the latter case the evacuation was accompanied by no pain nor constitutional disturbance, and she soon regained her natural health, and is now perfectly well.

The author observes that the remarkable circumstance in this case is, that the same phenomena should occur in two consecutive pregnancies, an interval of between four and five years having elapsed between them: he also says that it is the first case of the kind that has been observed, as he cannot find a similar one recorded in the annals of medicine.

[As Dr. Galiay says, the preceding case derives the whole of its interest, from occurring twice in succession and running through the same course. We have not been able to find any other recorded which exactly resembles it. In the 5th vol. of the Edin. Med. Essays there is related a case, in which the patient seemed to have a second extra-uterine pregnancy before she got rid of the first. Several instances have been met with where a woman has conceived again, and even borne a living child, while a former fœtus has been retained in the abdomen. It is a matter of regret that the author has not detailed this case with greater precision and fulness. The exact time intervening between the different periods should have been mentioned, as well as the state of the remains of the fœtuses at the time of their expulsion; whether any purulent or putrid matter was evacuated along with the bones, which are the only parts mentioned; and whether the fragments of the second fœtus were in the same state as those of the first.]

*Gazette Médicale de Paris. July* 29, 1837.

